# The Effect of Transformational Leadership, Servant Leadership, and Organizational Learning on Manufacturing Industry Performance

**DOI:** 10.3389/fpsyg.2022.895361

**Published:** 2022-05-09

**Authors:** Francisca Sestri Goestjahjanti, Sahala Benny Pasaribu, Tatang Iman Sadewo, Srinita Srinita, Isyak Meirobie, Agustinus Purna Irawan

**Affiliations:** ^1^Department of Economics and Management, Sekolah Tinggi Ilmu Ekonomi Insan Pembangunan, Tangerang, Indonesia; ^2^Faculty of Economics and Business, Trilogi University, South Jakarta, Indonesia; ^3^Faculty of Economics and Business, Syiah Kuala University, Banda Aceh, Indonesia; ^4^Faculty of Economics and Business, Tarumanagara University, West Jakarta, Indonesia; ^5^Faculty of Engineering, Tarumanagara University, West Jakarta, Indonesia

**Keywords:** transformational leadership, servant leadership, organizational learning, performance, manufacturing industry

## Introduction

In the era of the industrial revolution 4.0, a good manufacturing industry (MI) has a vision and mission to achieve the goals to be accomplished. It would be better if the MI experience a continuous improvement and development. One of the improvements that the manufacturing industry strives for is to increase or maintain the advantages it possesses. According to Birasnav et al. (2013), Crawford (2003), and Desky et al. (2020), the competition in today's business world is getting tougher, and the MI is required to survive in this competition. The influence of leadership on the company also varies. One of the important things that companies can do to survive in the intense competition is to improve their company's performance by developing and improving the company's leadership style. Another factor of the performance of a company is servant leadership. The leaders who are most able to motivate their followers are the leaders who are not maximal in satisfying their own needs, but mostly in prioritizing to meeting the needs of their followers. According to Degrees et al. (2014), Gessler and Ashmawy (2016), and Haudi et al. (2022), the employees who have servant leadership type of leaders are more likely to be motivated to make new innovations to achieve the results expected by the company. Meanwhile, the MI learning is a set of behaviors related to the industry that show a commitment to learning and continuously making improvements. According to Hulpia et al. (2011) and Kadiyono et al. (2020), the MI learning is a type of activity where the MI learns. If employees have the willingness to learn to achieve better results than the previous results, the resulting MI performance will be at its maximum.

The research on the performance of the MI has been studied by Hulpia et al. (2011), Kadiyono et al. (2020), and Purwanto et al. (2021a,b), who examined the effect of transformational leadership on the performance of the MI through MI learning and innovation, showing that the research variables are interrelated. The positive aspects include transformational leadership, MI learning, MI innovation, and performance. The research was also conducted by Ojokuku et al. (2012), which showed that the charismatic leadership style and the bureaucratic leadership style had a negative effect on the performance of the MI. The transactional leadership styles and autocratic leadership styles have no effect on MI performance. The transformational leadership has a positive effect on the performance of the MI. Similar research was also conducted by Samad (2012), showing that innovation and transformational leadership are positively related to the performance of the MI. The research conducted by Choudhary et al. (2013) shows that the transformational leadership and servant leadership have a positive effect on learning in the MI. Learning in the MI has a positive effect on the performance of the MI. Another study was also conducted by Noruzy et al. (2013) and showed that the transformational leadership is positively related to MI learning, knowledge management, manufacturing industry innovation, and MI performance. The next result is that the MI learning is positively related to the knowledge management, MI innovation, and MI performance. Furthermore, the knowledge management and innovation in the MI are positively related to the performance of the MI (Fahlevi, 2021).

According Gelard et al. (2014) the aspect of the transformational leadership is that leaders can motivate followers to carry out tasks above expectations and beyond their own self-interest, for the benefit of the MI. Transformational leaders have the characteristics of charisma (providing a vision and mission, instilling pride, and gaining respect and trust), inspiration (communicating high expectations, using symbols to focus efforts, and expressing important goals in a simple way), intellectual stimulation (encourage intelligence, rationality, and problem solving), and individual judgment (giving personal attention, treating each employee individually, and training and advising the subordinates). According to Sunarsi et al. (2020), Supriadi et al. (2020), Quddus et al. (2020), and Purwanto et al. (2020), transformational leadership is a leader who can make changes to employees and can inspire employees; for example, by motivating their employees. Based on the description above, the authors make the following hypothesis:

H1: Transformational leadership has a positive effect on learning in the MI.

**Figure 1:**
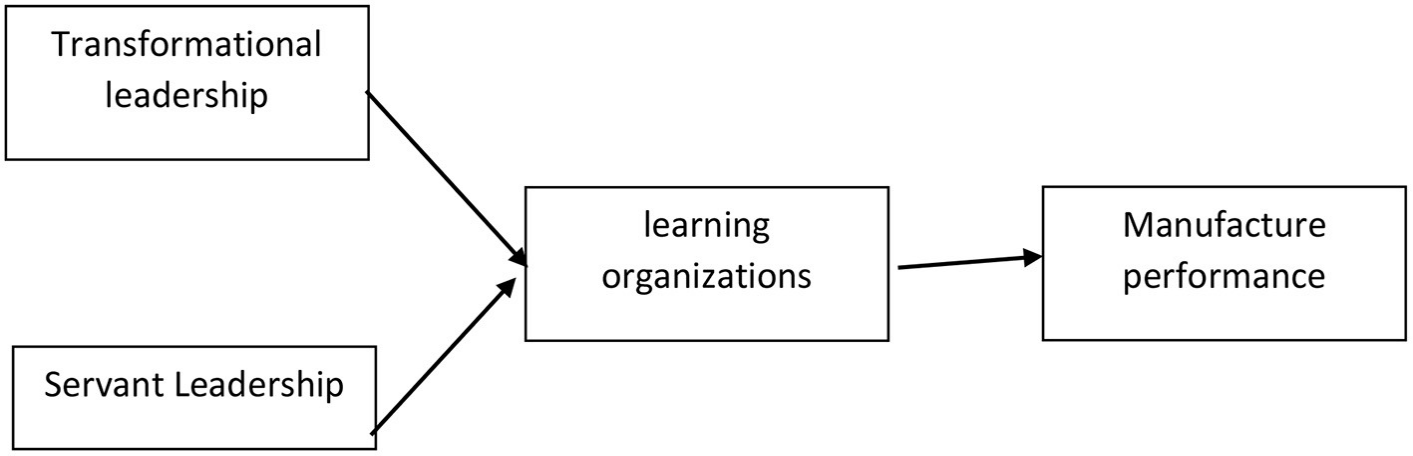
Theoretical framework.

Employees are seen as one of the greatest assets of the MI. Retaining loyal and productive employees and increasing profits is a challenge for leaders, as is encouraging an understanding of employee engagement. According to Tas (2017) and Suyitno et al. (2014), the servant leadership is based on the premise that leaders who are most able to motivate followers are leaders who are not optimal in satisfying their own needs and who mostly prioritize meeting the needs of their followers. As a result, the servant leadership is defined as a leader who is more focused on improving the MI's attitude, ethics, and culture. For example, obeying the rules in the office, which include obeying the rules of working hours and how to dress. Based on the description above, the authors propose the following hypothesis in Figure 1:

H2: Servant leadership has a positive effect on learning in the MI.

The MI learning is based on the basic principles of learning, namely, receiving and collecting information, interpreting it, and acting on the interpretation of the information. Learning MI is a concept that provides a MI with the strength it needs to survive in the face of environmental changes. The application of the MI's learning makes the MI have the flexibility to adapt to environmental changes that are increasingly dynamic and difficult to predict. The MI Learning can also be described as a set of MI behaviors that show a commitment to learning and continuously making improvements. The MI Learning is a type of activity in the MI where the MI continues to learn, according to Tas (2017), Suyitno et al. (2014), Suharto et al. (2022), and Vizano et al. (2020). The MI learning is the behavior of employees who are committed to exchanging the information and learning to be even better in the future. Based on the description here, the researcher proposes the following hypothesis:

H3: The MI learning has a positive effect on MI performance.

## Methods

This study uses transformational leadership as an independent variable, servant leadership, and learning in the MI, while the dependent variable is the performance of the MI. The population in this study were employees of the MI. The sample used comprised of 400 employees from the MI. The sampling in this study used a non-probability sampling design with a convenience sampling method. To obtain the necessary data, the researcher distributed questionnaires. A total of 600 questionnaires were distributed. The questionnaires received among them and used in this study were 400 questionnaires. Each statement item used was measured using a Likert Scale with five alternative choices, namely, Strongly Disagree (STS), Disagree (TS), Neutral (N), Agree (S), and Strongly Agree (SS). The independent variables in this study consisted of transformational leadership, servant leadership, MI learning, and MI performance.

### Transformational Leadership

For the benefit of the MI, the leadership that inspires subordinates to go beyond their own desires is essential. The transformational leaders have a tremendous influence on their followers. The aspects of transformational leadership are leaders who can motivate followers to perform above expectations and beyond their own interests, for the sake of the MI. The measurement of the transformational leadership in this study uses four statement items adopted from Degrees et al. (2014), Gessler and Ashmawy (2016), and Haudi et al. (2022).

### Servant Leadership

The servant leadership is leadership that addresses ethical issues, customer experience, and employee engagement while creating a unique MI culture where leaders and followers come together to achieve MI goals without positional or authoritative power. Employees are seen as one of the greatest assets of the MI, retaining loyal and productive employees. Increasing profits and employee engagement is a challenge for the leaders. The measurement of servant leadership in this study uses nine statement items adopted from according to Degrees et al. (2014), Gessler and Ashmawy (2016), and Haudi et al. (2022).

### The MI Education

The MI Learning is described as a set of MI behaviors that demonstrate a commitment to learning and continuous improvement. The MI Learning is a type of activity in the MI where the MI continues to learn (Örtenblad, 2001). The measurement of MI Learning in this study uses four statement items adopted from According to Gessler and Ashmawy (2016) and Haudi et al. (2022).

### Dependent Variable

The MI performance is defined as the MI's ability to achieve goals effectively and efficiently by using existing resources. The measurement of MI performance in this study uses four statement items adopted from According to Sunarsi et al. (2020), Supriadi et al. (2020), and Quddus et al. (2020). Figure 1 describes the research model.

From Figure 1, it can be seen the relationship between the variables in this opinion research which was initiated as a form of a new model of leadership in the MI.

## Result and Discussion

The validity testing was carried out on four main variables in this study, namely, transformational leadership, servant leadership, MI learning, and MI performance. The results of the validity test of transformational leadership statement items are four statement items. The servant leadership variable has nine statement items, indicating that the remaining eight statement items have good validity. The MI learning variable consists of four statement items. The remaining two questionnaire items have good validity. The MI performance variable consists of four statement items. The remaining three statement items have good validity. All values have good validity. The condition is that the factor loading value is above 0.50. In the reliability test, the following results were obtained: the reliability of Cronbach's transformational leadership was 0.895; servant leadership was 0.870; MI learning was 0.975; and the MI performance was 0.980. All variables are reliable, with a Cronbach alpha value above 0.6.

The influence of transformational leadership on learning in the MI has a sig value of 0.009 <0.05 with a positive estimate value. This means that transformational leadership has a positive effect on learning in the MI, so it can be concluded that H1 is supported. This result is possible because restaurant leaders are always looking for new opportunities to improve yield and quality on new restaurant menus. This will result in mutual success in the MI as well as for an individual employee, because with superiors who are always looking for new opportunities or innovating, it will lead to the commitment of employees to exchange information and learn to improve the quality of menus in restaurants, which means that the MI learning that was applied was in accordance with what was expected by the restaurant. The results of this study support the research conducted by Choudhary et al. (2013), Suyudi et al. (2020), and Noruzy et al. (2013), with results showing that transformational leadership has a positive effect on learning in the MI.

The influence of servant leadership on learning in the MI has a sig value of 0.009 <0.05, with a positive estimate value. This means that servant leadership has a positive effect on learning in the MI, so it can be concluded that H2 is supported. This result is possible when employees are led by leaders with a servant leadership style. These employees will tend to be motivated to continue to make improvements to their performance, and employees are involved in creating a culture of learning together with other employees. This is because servant leadership considers employees as one of the biggest assets in the MI, so they need to be properly facilitated in order to develop more broadly in terms of their abilities, knowledge, and skills. The results of this study support research conducted by Hulpia et al. (2011), Kadiyono et al. (2020), and Purwanto et al. (2021a,b), with results showing that servant leadership has a positive effect on learning in the MI.

The influence of MI learning on MI performance has a sig value of 0.000 0.05 with a positive estimated value. This means that the learning of the MI has a positive effect on the performance of the MI, so it can be concluded that H3 is supported. This result is possible because if the restaurant has acquired and used a lot of new and relevant knowledge to use, it will create a competitive advantage. This can have an impact on the performance of the MI, such as increasing profits or assets over time. This will be meaning positive for the survival of restaurants in the long term or in the future. The results of this study support research conducted by Hulpia et al. (2011), Kadiyono et al. (2020), and Purwanto et al. (2021a,b), with results showing that MI learning has an effect positive on the performance of the MI.

## Conclusion

Based on the research that has been conducted on employees of the MI, it can be concluded that the transformational leadership has a positive effect on learning in the IM, servant leadership has a positive effect on learning in the MI and learning in the MI has a positive effect on the performance of the MI. Improvement of the performance of the MI in restaurants can be done by paying attention to and improving forms of leadership such as transformational leadership, servant leadership, and learning about the MI.

## Author Contributions

FG conceived of the idea presented here. SP and TS developed the theory and performed the computations. SS verified the analytical methods. IM encouraged AI to investigate the topic and supervised the findings of this work. All authors discussed the results and contributed to the final manuscript.

## Conflict of Interest

The authors declare that the research was conducted in the absence of any commercial or financial relationships that could be construed as a potential conflict of interest.

## Publisher's Note

All claims expressed in this article are solely those of the authors and do not necessarily represent those of their affiliated organizations, or those of the publisher, the editors and the reviewers. Any product that may be evaluated in this article, or claim that may be made by its manufacturer, is not guaranteed or endorsed by the publisher.
